# The Role of Auxin Transport in Plant Patterning Mechanisms

**DOI:** 10.1371/journal.pbio.0060323

**Published:** 2008-12-16

**Authors:** Richard S Smith

## Abstract

In plants, many patterning processes involve the phytohormone auxin, and controlling how it moves around plays a critical role in pattern formation.

## How Do Organisms Pattern Themselves?

One of the fundamental questions in developmental biology is how a multicellular organism is able to create complex forms and patterns, given that every cell has the same genetic code or program. A mouse embryo separated at the two-cell stage can give rise to two identical adults, but if left together, these same two cells somehow “know” about each other and develop into a single organism. The question of how a group of equivalent cells self-organize to create a pattern was addressed by Turing [1]. Using mathematical analysis, and what is perhaps the first example of computer simulation in developmental biology, Turing showed that a pattern could emerge from a group of identical cells, all operating with identical rules. These rules involved equations for the reactions of substances, which he termed morphogens, combined with diffusion. His reaction–diffusion model of morphogenesis has since become one of the most widely used models to explain pattern formation in biological systems [2,3].

Gierer and Meinhardt [4] developed a version of Turing's model that provides intuitive insight into how reaction–diffusion works. They noticed that in order for patterning to occur, there must be some element of local activation combined with a longer-range inhibition. The local activation selects particular cells for differentiation, whereas the longer-range inhibition is required to suppress the activation of neighbors. Cast in terms of abstract but plausible substances, the model was constructed as follows.

There are two substances, an activator and an inhibitor. The activator enhances its own production as well as the production of the inhibitor. The inhibitor, in turn, inhibits production of the activator. Such a system is easy to envision as a feedback loop in a genetic regulatory network (Figure 1). A slightly increased concentration of the activator in a cell due to random variation can lead to a small local increase in production of both the activator and the inhibitor in the cell. If the inhibitor diffuses to neighbor cells much more quickly than the activator, it will reduce the inhibitor's negative effect on local activator self-enhancement, and suppress the activation of cells nearby. In a system of identical cells, each operating with these same rules, a small amount of noise can lead to a periodic pattern of peaks high in activator concentration. This then triggers differentiation, leading to visible pattern formation. Meinhardt proposed and simulated many variants on this basic idea, and was able to reproduce a wide variety of the patterns observed in nature [2,5]. Now that molecular data have become available, these types of models are increasingly being cast in terms of real substances, belonging to real genetic regulatory networks [6–8].

**Figure 1:**
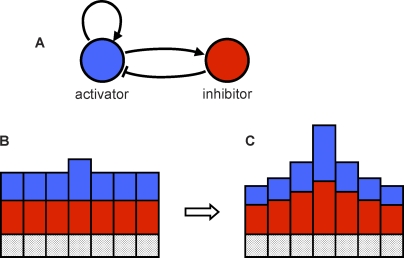
Activator–Inhibitor System The activator is shown in blue, and the inhibitor is shown in red. (A) The activator enhances its own production as well as the production of the inhibitor. The inhibitor inhibits production of the activator. (B and C) A line of cells, with the height of the blue and red bars indicating activator and inhibitor concentration. A slight increase in the concentration of the activator in one cell causes an increase in production of both the activator and the inhibitor in that cell (B). The inhibitor diffuses away more quickly than the activator, allowing local activation to escalate, while simultaneously suppressing neighbor cells (C).

## A Central Role for Transport in the Patterning of Plants

The plant hormone auxin is at the center of many key patterning events in plants. The selection of cells for organ initiation in the shoot apex, leaf venation, apical dominance, tropisms, and embryo axis formation are all under the control of auxin [9–12]. However, auxin is different from the morphogens considered by Turing. Instead of moving primarily via diffusion, it is actively pumped from cell to cell by the action of import and export proteins (Figure 2). Experimental evidence now starting to accumulate suggests that it is the control of auxin transport by these transport proteins that is central to many patterning processes involving auxin [13–15].

**Figure 2:**
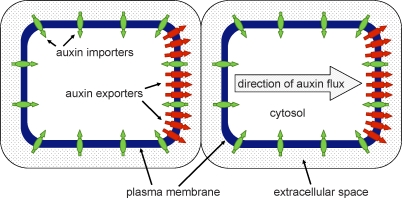
Auxin Transport Proteins in Arabidopsis Cells Export (red) and import (green) proteins sit in the plasma membrane and transfer auxin between the cytosol and extracellular space. Often, the export proteins are polarly localized, resulting in a net flux of auxin through the cells.

In the center of the meristem at the tip of the shoot are the so-called plant stem cells, which are surrounded by a small band of cells that are competent to initiate leaf or flower organs [16,17]. Experimental evidence suggests that organs initiate in this zone in response to auxin maxima, which are created by the coordinated transport of auxin to convergence points. In the surface layer of cells in the shoot apex, the auxin export protein, PIN1, localizes in the plasma membrane at one side of the cell, pumping auxin toward the sites where auxin accumulates [14,18]. Based on these results, Reinhardt et al. proposed a model for organ initiation in which the local activation of cells is not caused by local self-enhanced production, as is the case in reaction–diffusion models, but rather by the directed transport of auxin to organ initiation sites (Figure 3). Longer-range inhibition does not require a second substance; it is due to the removal of auxin from surrounding tissue. But this in itself does not provide a complete mechanism for patterning, as it does not answer the question as to what controls the orientation of the PIN1 transport proteins. A possible answer to this question came from computer simulation studies [19,20] in which it was hypothesized that PIN1 proteins are able to react to the concentration of auxin in neighbor cells, and orient preferentially toward cells with higher concentration. Similar in concept to reaction–diffusion, if one cell has a slightly higher auxin concentration, then this causes the PIN1 proteins in neighboring cells to orient preferentially toward it, causing a further increase in concentration. In a tissue of cells, this can result in a spacing mechanism similar to Meinhardt and Gierer's activator–inhibitor system.

**Figure 3:**
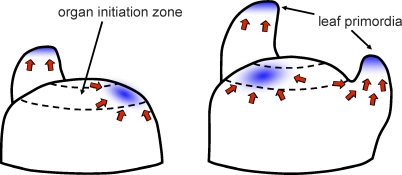
Auxin Transport Model of Organ Initiation in the Shoot Apex Auxin export proteins (red arrows) pump auxin toward auxin convergence points (blue) that cause organ initiation. Auxin is drained from surrounding tissue, inhibiting the formation of new primordia nearby. Adapted from [20].

Organ initiation in the shoot apex is not the first example of auxin feeding back on its own transport that has been suggested as a patterning mechanism in plants. In Sachs' canalization hypothesis for vein formation [21], auxin transport is thought to respond to auxin flux rather than concentration. This hypothesis proposes that the canalization of auxin into preferred routes of auxin flux occurs in much the same way that water carves rivers in soft terrain. A cell's ability to transport auxin is assumed to increase with auxin flux, causing any initially dominant path to be reinforced. As soon as the smallest canal begins to emerge due to random variation, it will be enhanced and attract even more flow. This causes the preferred path to strengthen (local activation), while suppressing alternate routes nearby (long-range inhibition). Simulation models of this patterning mechanism have shown that it is indeed capable of selecting strands of cells from a tissue of undifferentiated cells [22–24].

## Several New Twists on Transport-Based Patterning in the Root

The root meristem is responsible for the architecture of the subsurface portion of the plant. In Arabidopsis, the root meristem is in some ways a simpler and more ordered structure than the shoot meristem, yet its auxin transport dynamics are amazingly complex. A combination of experimental and simulation modeling techniques [25] suggest that the peak of auxin concentration at the center of the root tip is the result of a reflux system driven by the transport of auxin by various members of the PIN transport protein family. As with shoots, the root must also periodically create lateral organs to extend its structure, and the experimental data suggest that this process is also triggered by elevated auxin levels [26]. In the root, however, lateral root primordium initiation does not appear to be accompanied by dramatic changes in PIN relocation, as is the case with the patterning in leaves and shoots. Instead, an article by Laskowski et al. in this issue of *PLoS Biology* [27] suggests that it is the auxin import protein AUX1, combined with the geometry of the cells themselves, that is the crucial player in the patterning mechanism behind lateral root initiation.

In the models of organ initiation in the shoot, import proteins have been thought to play only a secondary role in patterning. They are essential for pattern formation, since organ initiation in the shoot is severely impaired when these proteins are knocked out [28]. However, they do not polarize to one side of the cell as PINs do, nor are they preferentially expressed at organ initiation sites. Laskowski et al. present data indicating that importers play a much more central role in the root. They show that the local auxin maximum associated with organ initiation in the root occurs concomitantly with an up-regulation of the AUX1 importer. Their model is based on the idea that this up-regulation would then cause the cells to retain even more auxin, leading to a self-enhancing activation of the cells (Figure 4). Inhibition of neighbor cells would similarly be caused by the depletion of auxin from surrounding tissue. In their model, the AUX1 import protein is the active player, with the PINs playing a necessary but more passive role.

**Figure 4:**
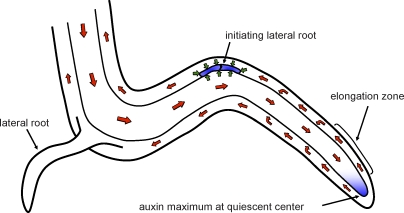
Auxin Transport Model of Lateral Root Initiation Polarly localized auxin export proteins (red arrows) pump auxin toward the quiescent center at the tip of the root, creating an auxin maximum there (blue). Auxin is then returned upwards by polarly localized auxin exporters in the outer layers of cells in the root. A portion of the auxin returns into the reflux stream due to lateral orientation of some of the exporters. In the model of Laskowski et al., bends in the root caused by anisotropic growth in the elongation zone induce a mini-reflux loop, causing auxin to accumulate preferentially on the outside of the curve. This induces cells to produce additional import carriers (small green arrows), which creates an auxin maximum (blue cells) by skimming auxin out of the transport stream. This auxin maximum then triggers lateral root formation.

To start the process, Laskowski et al. suggest that it is not simply noise, or distance from previous lateral root primordia, that gives select cells that initial slight advantage, but that it is the result of the geometry of the cells themselves. Using computer simulation they show that, under suitable conditions, the shape of the cells tends to favor auxin accumulation on the outside of a curve in the root. This slight advantage given by the shape initiates the self-enhancing activation of AUX1. The idea is conceptually attractive, as making a branch at a point where it would be possible to explore more soil area has obvious adaptive significance. But if the patterning is influenced by curvature, then it is natural to ask what causes this curvature. Although not fully understood, root waving, the alternating pattern of left–right curvature often observed in Arabidopsis seedlings grown on media, is thought to involve a combination of gravitropic and thigmotropic (mechanical contact) responses [29,30]. Thus the model suggests a link between mechanical events at the root tip, which cause changes in cell shape, and transport-based patterning events downstream. Mechanotransduction of signals in plants is usually thought of in terms of the direct transmission of the stresses themselves, or chemical signals that result from the activation of stress or strain receptors [29]. The work of Laskowski et al. adds an interesting geometrical twist to the ideas of mechanotransduction pathways and information storage in plants. In their model, information coming from the root tip that will ultimately help direct where the lateral root primordia initiate is stored in the geometry of the cells themselves.

## A Role for Simulation Modeling

The article by Laskowski et al. demonstrates the utility of a combined approach involving both experimental and computer simulation methods. It is not intuitively obvious that the shape of the cells in the transport stream of the root could cause auxin to preferentially accumulate on the outside of curves, initiating lateral roots. It is through simulation modeling that such ideas can be examined and tested, to see if they are plausible. This invariably leads to new questions, suggesting new directions for experimental work. Laskowski et al. use this synergy between experimentation and simulation to explore new mechanisms for plant patterning, setting the stage for further inquiry and experimental testing.
